# *In vivo* temporal and spatial profile of leukocyte adhesion and migration after experimental traumatic brain injury in mice

**DOI:** 10.1186/1742-2094-10-32

**Published:** 2013-02-28

**Authors:** Susanne M Schwarzmaier, Ricarda Zimmermann, Niamh B McGarry, Raimund Trabold, Seong-Woong Kim, Nikolaus Plesnila

**Affiliations:** 1 Institute for Surgical Research in the Walter-Brendel-Centre of Experimental Medicine, University of Munich Medical Center, Marchioninistr. 15, 81377 Munich, Germany; 2 Department of Neurodegeneration, Royal College of Surgeons in Ireland (RCSI), 123 St. Stephen’s Green, Dublin 2, Ireland; 3 Institute for Stroke and Dementia Research (ISD), University of Munich Medical Center, Max-Lebsche Platz 30, Munich, 81377, Germany; 4 Current address: Department of Anesthesiology, University of Munich Medical Center, Marchioninistr. 15, 81377 Munich, Germany

**Keywords:** Leukocytes, Inflammation, Brain trauma, Secondary brain damage, Leukocyte adhesion, Leukocyte-platelet aggregates, 2-photon microscopy, Intravital microscopy, *In vivo*, Mice

## Abstract

**Background:**

Leukocytes are believed to be involved in delayed cell death following traumatic brain injury (TBI). However, data demonstrating that blood-borne inflammatory cells are present in the injured brain prior to the onset of secondary brain damage have been inconclusive. We therefore investigated both the interaction between leukocytes and the cerebrovascular endothelium using *in vivo* imaging and the accumulation of leukocytes in the penumbra following experimentally induced TBI.

**Methods:**

Experimental TBI was induced in C57/Bl6 mice (n = 42) using the controlled cortical impact (CCI) injury model, and leukocyte-endothelium interactions (LEI) were quantified using both intravital fluorescence microscopy (IVM) of superficial vessels and 2-photon microscopy of cortical vessels for up to 14 h post-CCI. In a separate experimental group, leukocyte accumulation and secondary lesion expansion were analyzed in mice that were sacrificed 15 min, 2, 6, 12, 24, or 48 h after CCI (n = 48). Finally, leukocyte adhesion was blocked with anti-CD18 antibodies, and the effects on LEI and secondary lesion expansion were determined 16 (n = 12) and 24 h (n = 21), respectively, following TBI.

**Results:**

One hour after TBI leukocytes and leukocyte-platelet aggregates started to roll on the endothelium of pial venules, whereas no significant LEI were observed in pial arterioles or in sham-operated mice. With a delay of >4 h, leukocytes and aggregates did also firmly adhere to the venular endothelium. In deep cortical vessels (250 μm) LEIs were much less pronounced. Transmigration of leukocytes into the brain parenchyma only became significant after the tissue became necrotic. Treatment with anti-CD18 antibodies reduced adhesion by 65%; however, this treatment had no effect on secondary lesion expansion.

**Conclusions:**

LEI occurred primarily in pial venules, whereas little or no LEI occurred in arterioles or deep cortical vessels. Inhibiting LEI did not affect secondary lesion expansion. Importantly, the majority of migrating leukocytes entered the injured brain parenchyma only after the tissue became necrotic. Our results therefore suggest that neither intravascular leukocyte adhesion nor the migration of leukocytes into cerebral tissue play a significant role in the development of secondary lesion expansion following TBI.

## Introduction

Leukocytes are believed to play an important role in secondary brain damage following acute brain injury such as stroke or brain trauma [1]. Following brain injury, blood-borne leukocytes begin to roll on - and subsequently stick to - the cerebrovascular endothelium, and then finally migrate into the cerebral tissue, where they are believed to cause damage, e.g. by releasing reactive oxygen species [2]. Inhibiting the adhesion of leukocytes to the endothelium (for example, by blocking intercellular adhesion molecule 1 (ICAM-1) or MAC-1), resulted in a significant reduction in infarct volume in experimental models of focal and global cerebral ischemia [3-7].

A similar sequence of events also seems to occur following traumatic brain injury (TBI); however, the role of leukocyte invasion in secondary brain damage remains somewhat controversial. Several reports support a contributing role of leukocytes in secondary brain damage. For example, an accumulation of polymorphonuclear leukocytes in the brain correlated with increased intracranial pressure (ICP) and reduced cerebral blood flow (CBF) following cold injury in rats [8]. Antibodies directed against leukocyte adhesion molecules (for example, anti-ICAM-1) reduced leukocyte accumulation in the tissue and led to improved neurological function following fluid percussion injury (FPI) [9]. Mice deficient in T and B cells and mice that were treated with T cell inhibitory agents had less traumatic brain damage following aseptic cerebral injury (ACI) than controls [10]. A 50% reduction in total post-FPI contusion volume was correlated with a reduction in the number of accumulating monocytes/macrophages in the medial cortex three days after injury [11]. Finally, neutrophil depletion reduced CCI-induced edema formation and contusion volume in mice [12].

On the other hand, several studies have reported that neither inhibiting leukocyte adhesion with anti-CD18 antibodies nor depleting neutrophils affected the permeability of the blood–brain barrier (BBB) following experimental TBI [13-15], and mice that were deficient in P-selectin and ICAM-1 exhibited neuroprotection without a change in leukocyte accumulation [16]. Additionally, most studies regarding the role of leukocytes in post-traumatic brain damage did not investigate intravascular leukocyte accumulation, nor did they correlate the spatial and temporal accumulation of leukocytes in traumatized brain tissue. Accordingly, it remains unclear whether leukocytes adhere to the cerebrovascular endothelium, migrate into damaged tissue, and cause additional damage or whether they migrate into the damaged brain tissue only after secondary brain injury has occurred. To address these questions, we investigated both the time course and the effect on secondary contusion growth of a) leukocytes that accumulate in the tissue, and b) intravascular leukocytes and leukocyte-platelet aggregates that adhere to the cerebrovascular endothelium following traumatic brain injury precisely in the region in which secondary brain damage occurs.

## Material and methods

### Animals

For this study, we used 6 to 8-week-old male C57Bl6 mice (23 to 26 g) that were obtained from either Charles River (Kisslegg, Germany) or Jackson Laboratories (Bicester, UK). The animals had free access to tap water and pellet food. Mice that were allowed to awaken between subsequent procedures within one experiment were housed individually throughout the experiment. All animal experiments were conducted in accordance with institutional guidelines and approved by the government of Upper Bavaria (license number and ethical approval number 06/04), and by both the Ministry for Health and Children in Dublin, Ireland (license number B100/4169) and the Research Ethics Committee of the Royal College of Surgeons (REC number 467).

### Controlled cortical impact

Traumatic brain injury was induced in a large craniotomy window over the right hemisphere using a controlled cortical impact (CCI) device that was optimized for use in mice [17,18]. For experiments involving intravital microscopy or histological assessment, the impact piston travelled at 6.0 or 8 m/s, respectively, with a penetration depth of 0.5 or 1.0 mm, respectively; the contact time with the tissue was 150 ms. Thereafter, the cranial bone was re-implanted and affixed with histoacrylic glue. Sham-operated animals were subjected to the same surgical procedure without the induction of a trauma.

### Experimental protocol for intravital microscopy monitoring

These experiments were performed as previously described [18-20]. In brief, animals were anesthetized by an intra-peritoneal injection of medetomidine (0.5 mg/kg, Domitor®), fentanyl (0.05 mg/kg), and midazolam (5 mg/kg, Dormicum®). Following induction, the mice were endotracheally intubated and ventilated using a volume-controlled ventilator. Body temperature, end-tidal CO_2_ and invasive systolic blood pressure were monitored continuously.

**Figure 1:**
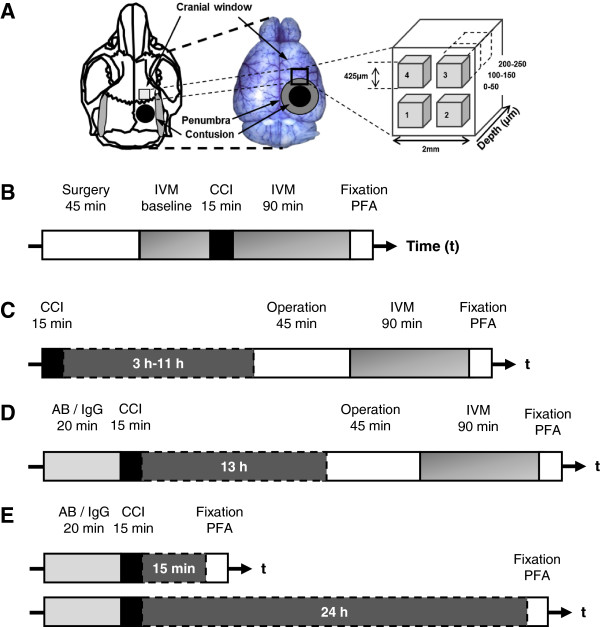
**Experimental design. **(**A**) Schematic illustration of the imaging window and controlled cortical impact (CCI)-induced contusion and penumbra sites. (**B**) Timeline of the experiments for monitoring the cerebral microcirculation, including baseline recording. (**C**) For observations up to 14 h post-trauma, the mice were allowed to recover in their respective cages until the preparation for intravital microscopy (IVM). (**D**) Following an injection of either anti-CD18 antibodies or control IgG, the animals were subjected to CCI; 13 h thereafter, the mice were prepared for IVM. All IVM experiments ended with perfusion-fixation with paraformaldehyde and the subsequent removal of the brain. (**E**) The animals received either control IgG or anti-CD18 antibodies and were then subjected to CCI; 15 min or 24 h later, their brains were removed for histological assessment.

Subsequently, the animals were immobilized in a stereotactic frame, and two cranial windows - one for TBI and one for IVM monitoring - were prepared over the right hemisphere. After two baseline recordings of selected cerebral arterioles and venules, the animals were subjected to either TBI or a sham operation (n = 6 mice per group); the animals were then transferred back to the intravital microscope, and the previously observed vessels were re-monitored 30, 60, 90, and 120 min after CCI (Figure 1A + B). To monitor LEI at later time points (4 to 5.5 h, 8 to 9.5 h, and 12 to 13.5 h post-CCI; n = 6 animals per time point) mice were anesthetized with 2% isofluorane in 65% N_2_O and 33% O_2_ and subjected to CCI as described above. Subsequently, the animals were allowed to awaken in a recovery chamber (heated to 33°C and containing 50% humidity). Upon the recovery of motor function, the mice were transferred to their respective cages. After 3, 7, or 11 h, the animals were prepared for *in vivo* imaging as described above, and vessels were observed four times every 30 min (see Figure 1C). At the end of each experiment, the animals were sacrificed by transcardiac perfusion with 4% PFA.

### Intravital microscopy of superficial vessels

A square (2 mm × 2 mm) cranial window was prepared over the right fronto-parietal cortex; the dura mater was kept intact. The cerebral microcirculation was then investigated in an area 1.5 to 3.5 mm frontal to the primary contusion (Figure 1A), that is, in the region in which secondary brain damage occurs [18,21]. The animals were placed on a computer-controlled microscope stage for repeated analyses of the same vessels. Visualization of the microvessels was facilitated by an intravenous injection of fluorescein isothiocyanate-labeled dextran (FITC-dextran; 0.1 ml of a 0.5% solution; molecular weight 150,000; Sigma Chemical, St. Louis, Missouri, USA). Before each measurement, leukocytes were stained by repeated intravenous injections of the fluorescent dye rhodamine 6 G (0.05 ml of a 0.01% solution; Merck, Darmstadt, Germany). The images were collected using a video camera and recorded on videotape.

### Analysis of leukocyte-endothelium interactions in pial vessels

A computer-assisted microcirculation analysis system (CapImage; Ingenieurbüro Dr. Zeintl, Heidelberg, Germany) was used to quantify the IVM images off line by a frame-to-frame analysis [18]. The number of rolling and adherent leukocytes (7 to 12 μm in size) and aggregates (15 to 25 μm in size) was analyzed in the arterioles and venules by an investigator who was blinded with respect to the treatment of the animals. For each region of interest, a vessel segment 50 μm in length was studied for 30 sec in each measurement.

*Rolling* leukocytes/aggregates were identified by multiple intermittent contacts with the vascular endothelium and by their significantly lower velocity compared to freely moving leukocytes/aggregates in the central flow of the vessel. Leukocytes/aggregates were categorized as *adherent* when they attached firmly to the vascular endothelium for longer than 30 sec.

### Intravital microscopy of vessels in deeper regions of the brain

To investigate LEI in deeper regions of the brain (that is, at a depth of 50, 150, and 250 μm), we used a Zeiss 2-photon imaging system that was based on an LSM 710 confocal microscope equipped with a Chameleon Vision ΙΙ Ti:Sa laser (Coherent Scotland Limited, West of Scotland Science Park, Glasgow, Scotland). The principle of multi-photon microscopy implies that there is only one point where the excitation photons meet and thus result in an excitation of the dye. The great advantage of this technique is that this point can be at any chosen level within several 100 μm depths in the tissue. The emitted light will always come from this one point and can be detected with high spatial accuracy [22]. The animals were subjected to CCI and prepared for *in vivo* imaging as described above. For 2-photon imaging, a 2 mm × 2 mm craniotomy window was prepared under continuous cooling with saline, the dura mater was carefully removed, and a custom-made cover glass (Schott Displayglas, Jena, Germany) was inserted and affixed with dental cement (Cyano Veneer, Hager & Werken, Duisburg, Germany).

Eight to ten hours after CCI, animals were transferred to the 2-photon microscope and IVM was performed according to a standardized protocol. The cranial window was divided equally into four parts, each containing one region of interest (ROI). In total, we monitored 12 regions (that is, four ROIs at three different depths). Each ROI covered a volume of 425 μm × 425 μm × 50 μm, and the layers covered 0 to 50 μm, 100 to 150 μm, and 200 to 250 μm below the surface see Figure 1A. Z-stacks were acquired in 2-μm steps within 30 sec. For each ROI, two consecutive z-stacks were acquired. Vessels, leukocytes, and leukocyte-platelet aggregates were labeled as described above. We used an excitation wavelength of 830 nm and detected green and red fluorescence using two non-descanned detectors (NDD; BP500-550 and BP565-610).

### Analysis of leukocyte-endothelium interaction in deep vessels

The data were analyzed using ImageJ (downloaded from http://imagej.nih.gov/ij/) using the plugins *Calculator Plus, Co-localization,* and *3D Object Counter*. Leukocytes and leukocyte-platelet aggregates were identified according to their respective sizes as described above. By acquiring two consecutive images within 60 sec, we were able to identify leukocytes and aggregates as being either rolling or adherent.

### Contusion volume

The animals were deeply anesthetized with 4% isofluorane in 33% O_2_ and 63% N_2_O and sacrificed by cervical dislocation 15 min or 2, 6, 12, or 24 h after CCI. Subsequently, the brain was removed, snap-frozen on powdered dry ice, and stored at −80°C. Coronal sections (10-μm thick) were collected every 500 μm through the entire brain using a cryostat, stained with cresyl violet, and digitally recorded. The area of the contusion and of both hemispheres was quantified using an image analysis system (Olympus DP-SOFT; Olympus, Hamburg, Germany) by an investigator who was blinded with respect to the treatment conditions. Compared to healthy tissue the nuclei in the contusion are pyknotic and densely stained and the neuropil is very pale [21,23,24].

The size of the necrotic areas was corrected for brain swelling, and total contusion volume was calculated based on the contusion areas that were obtained from 15 sections [17,21].

### Immunohistochemistry

Staining was performed on frozen or paraffin-embedded tissues as described previously [25]. Paraffin sections were used to detect CD45-positive leukocytes and CD5 positive T lymphocytes; frozen sections were stained for the detection of Ly6G-positive granulocytes and monocytes, CD19-positive B lymphocytes and CD3ε-or TCRß- positive T lymphocytes. Before staining, the sections were fixed in methanol then air-dried, after which endogenous peroxidase activity was blocked with 3% H_2_O_2_ for 10 min. To label the leukocyte subpopulations, we used the following primary and secondary (horseradish peroxidase-conjugated) antibodies (all from BD Biosciences Pharmingen, California, USA): rat-anti-mouse-CD45 (Ly-5) antibody (1:50) followed by goat-anti-rat antibody (1:50); rat-anti-mouse-Ly6G and Ly6C (Gr-1) antibody (1:100) followed by goat-anti-rat antibody (1:50); rat-anti-mouse-CD19 antibody (1:500) followed by goat-anti-rat antibody (1:50); hamster-anti-mouse-CD3ε antibody (1:100) followed by goat-anti-hamster antibody (1:50); rat-anti-mouse-CD5 (Ly-1) antibody (1:50) followed by goat-anti-rat antibody (1:50); or hamster-anti-mouse-TCRß-chain antibody (1:3000) followed by mouse-anti-hamster antibody (1:50). The sections were incubated for 60 and 30 min with the primary and secondary antibodies, respectively. Between each step, the sections were rinsed twice for 10 min with PBS. Staining was visualized using a DAB detection kit according to the manufacturer’s instructions (VectorLabs, Burlingame, CA, USA). The nuclei were counter-stained with methylene blue. For positive controls, we used splenic tissue; for negative controls, some sections were not incubated with the primary antibody.

The section that was selected for quantification was the section that contained the largest contusion among 15 coronal sections that were cut through the contused brain. Leukocytes were quantified by counting all of the labeled cells in the injured and uninjured hemispheres. The analysis was performed by an investigator who was blinded with respect to the treatment of the animals. To assess post-trauma leukocyte accumulation in the brain, 48 animals were randomly assigned to the following six groups: 15 min and 2, 6, 12, 24, and 48 h following CCI. For each time point, paraffin and frozen sections were prepared from four animals.

### Inhibition of leukocyte adherence using anti-CD18 antibodies

Animals were anesthetized with 2% isofluorane in 65% N_2_O and 33% O_2_, then received 1.2 μg/g bodyweight of either an antibody directed against CD18 (GAME-46, BD Biosciences Pharmingen, California, USA) or a control IgG delivered via the femoral vein [26]. Subsequently, the animals were subjected to CCI as described above. To investigate the antibody’s effect on LEI, we performed IVM in both groups (n = 6 mice per group) at 16 and 16.5 h post-injury as described above (Figure 1D). To detect the potential effect of anti-CD18 treatment on secondary brain damage, animals were sacrificed 15 min or 24 h post-injury (n = 7 mice per group; Figure 1E), and contusion volume was assessed by histomorphometry as described above.

### Statistical analysis

Sample size was calculated before start of the study based on the following parameters: for the IVM experiments, a standard deviation of 25 to 30% of mean, a minimally detectable difference of at least 50%, and a power of 0.8; for histological assessment, a standard deviation of 15%, a minimally detectable difference of at least 25%, and a power of 0.8.

The Mann–Whitney *U*-test was used to analyze the differences between groups. The Friedman one-way analysis of variance on ranks followed by the Student-Newman-Keuls test was used to analyze differences over time. Data obtained from the histological analysis are presented as mean +/− standard deviation (SD), and data acquired from the *in vivo* imaging experiments are presented as mean +/− standard error of the mean (SEM), unless indicated otherwise. Differences with a *P*-value of <0.05 were considered to be statistically significant.

## Results

### Animal physiology

The blood gases, blood electrolytes, and physiological parameters (mean arterial blood pressure and core body temperature) of the animals were monitored and maintained within physiological limits throughout all experiments. Tables 1 and 2 show representative examples taken from two experimental groups. There were no significant differences between the groups or between different time points within an individual group for any physiological parameter.

**Table 1:** Physiological parameters of the animals at the indicated times during the experiments

	**16 hours**	**16.5 hours**	**Mean**
end-tidal pCO2 control IgG	33.6 +/− 2.1	32.8 +/− 1.9	33.2 +/− 1.9
end-tidal pCO2 anti-CD18 AB	32.8 +/− 1.2	31.0 +/− 1.0	31.9 +/− 1.1
MABP control IgG	74.0 +/− 7.2	69.6 +/− 3.4	71.8 +/− 5.2
MABP anti-CD18 AB	78.0 +/− 3.4	71.0 +/− 4.0	74.5 +/− 3.4
Ventilation f control IgG	149 +/− 8.4	144 +/− 8.3	146.5 +/− 8.1
Ventilation f anti-CD18 AB	141.6 +/− 8.8	145.0 +/− 8.8	143.3 +/− 8.5

**Table 2:** Arterial blood gases

	**pH**	**pCO2**	**pO2**
Final arteriolar blood gas control IgG	7.3 +/− 0.0	39.1 +/− 2.6	162.2 +/− 20.4
Final arteriolar blood gas anti-CD18 AB	7.2 +/− 0.0	44.3 +/− 3.2	141.4 +/− 24.9

Physiologically important parameters were monitored continuously throughout the experiments (n = 7 animals per group). End-tidal pCO_2_, mean arterial blood pressure (MABP) and ventilation frequency were recorded throughout every IVM recording experiment included in this study. The parameters remained within the physiological range as shown in this representative table of values obtained in the experiments testing the effect of anti-CD18 antibodies or IgG control. None of the parameters showed a significant difference between groups.

At the end of each IVM recording experiment blood gases were analyzed. The pH, pCO_2_ and pO_2_ remained within physiological thresholds and showed no significant difference between groups. The observed metabolic acidosis is not unusual for laboratory rodents fed with standard chow.

### Leukocyte-endothelium interactions in the superficial vessels 0 to 14 hours post-controlled cortical impact

**Figure 2:**
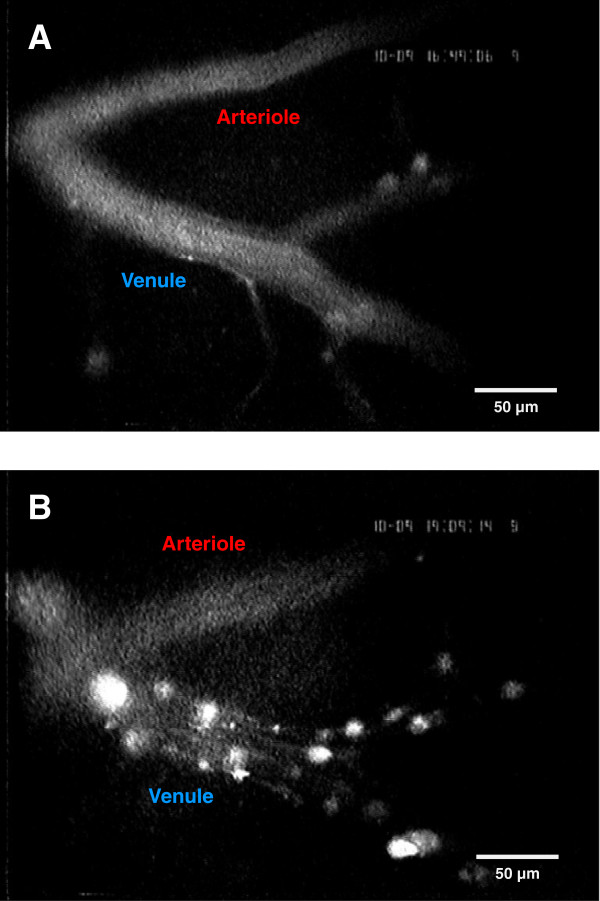
**Representative images of superficial vessels. **(**A**): A cerebral arteriole and a cerebral venule under baseline conditions. Neither adherent leukocytes nor a large number of rolling leukocytes are visible. (**B**): The same vessels as in A, 2 h after controlled cortical impact. Rolling and adherent leukocytes and aggregates are visible, predominantly in the venule.

**Figure 3:**
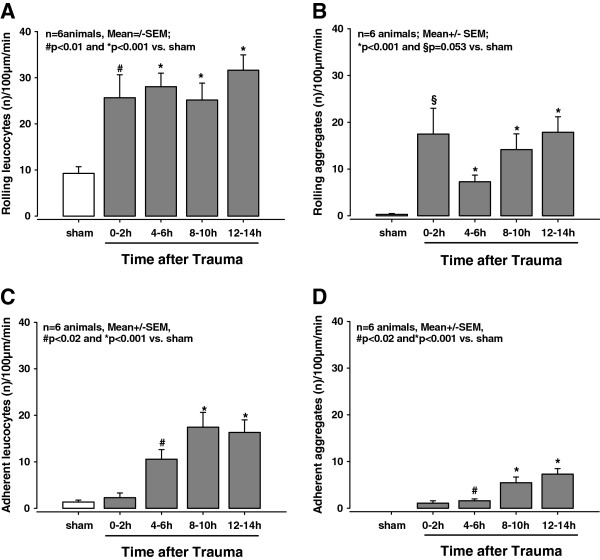
**Quantification of leukocyte-endothelium interaction (LEI) in superficial vessels. **(**A**- **C**) Number of rolling or adherent leukocytes in cerebral venules at the indicated times after controlled cortical impact (CCI). The number of rolling leukocytes was clearly increased immediately following CCI. The number of adherent leukocytes reached significance after 4 h. (**B,D**) The number of rolling and adherent aggregates in the cerebral venules after CCI showed a pattern similar to the leukocytes.

Under normal physiological conditions, leukocyte rolling occurs at a rate of 8.1 +/− 0.4 leukocytes/100 μm/min as quantified in the venules of the sham-operated mice and under baseline conditions prior to TBI. No other leukocyte interactions were observed in the venules or arterioles (Figure 2A; Figure 3A,C). Within two hours of inducing trauma, the number of rolling leukocytes in the venules increased significantly to 25.7 +/− 5/100 μm/min (*P < 0.01* versus baseline and sham; Figure 2B; Figure 3A). This enhanced level of LEI was stable until the end of the observation time, that is, 13.5 h after trauma (*P < 0.001* versus baseline and sham; Figure 3A).

The increase in the number of adherent leukocytes in the venules only reached significance 4 h following CCI (5 +/− 2.1 adherent leukocytes/100 μm/min), which was an increase of approximately ten-fold over baseline (*P < 0.02* versus baseline and sham). After 8 h, this number reached 17.5 +/− 3.2 adherent leukocytes/100 μm/min, and this rate was stable until the end of observation time (*P < 0.001* versus baseline and sham; Figure 3C). In arterioles, neither rolling nor adherent leukocytes were present in noteworthy numbers.

We also observed the formation of leukocyte-platelet aggregates, which were defined by their size of 15 to 25 μm [18]. No rolling or adherent aggregates were evident under baseline conditions in either the venules or arterioles (Figure 2A).

Following TBI, rolling aggregates appeared in the venules within the first two hours after injury (Figure 2B), although this did not reach significance (*P = 0.053* versus baseline and sham; Figure 3B). In contrast, at 4 h post-injury, the number of rolling aggregates had reached highly significant levels (7.3 +/− 1.4 rolling aggregates/100 μm/min; *P < 0.001* versus baseline and sham). Finally, we measured 17.9 +/− 3.3 rolling aggregates/100 μm/min at 13.5 h post-injury; the number of rolling aggregates had not reached a plateau by the end of the observation time (Figure 3B).

Similar to adherent leukocytes, the number of adherent aggregates only became significant at 4 h post-CCI (1.6 +/− 0.4/100 μm/min; *P < 0.01* versus baseline and sham). The number of adherent aggregates continued to increase over time without reaching a plateau (13.3 +/− 1.2 adherent aggregates/100 μm/min at 13.5 h post-trauma; *P < 0.001* versus baseline and sham; Figure 3D). We observed no rolling or adherent aggregates in the arterioles.

### Leukocyte-endothelium interactions at a depth of 0 to 250 μm 8 h post-controlled cortical impact

**Figure 4:**
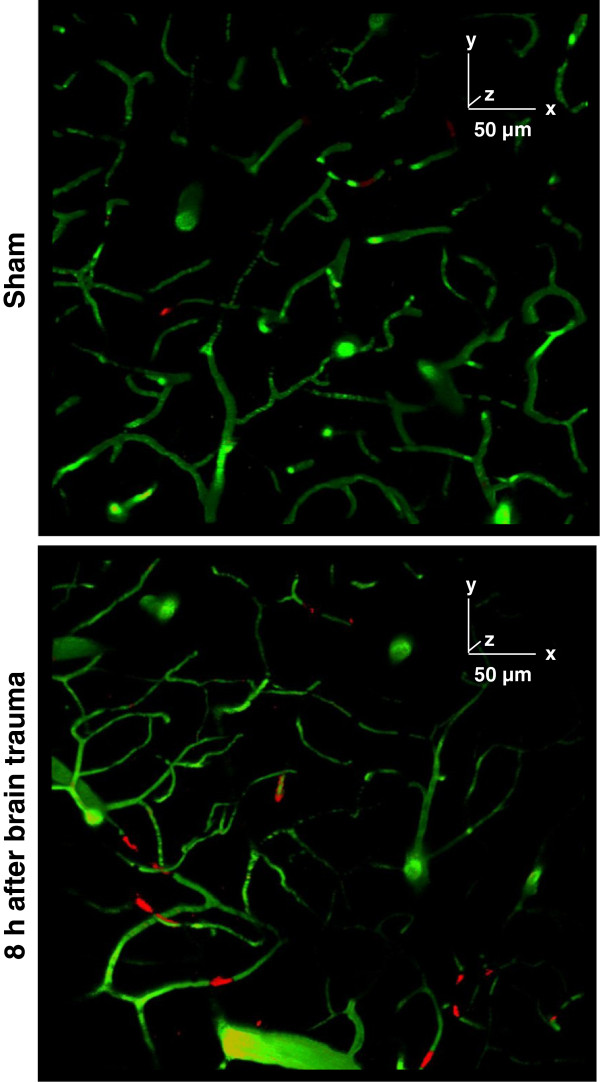
**Representative images of leukocyte-endothelium interaction (LEI) at a depth of 100 to 150 μm.** The vessel lumen is stained with fluorescein isothiocyanate-labeled (FITC) dextran (green), and leukocytes are labeled with rhodamine 6 G (red). Eight hours after controlled cortical impact (CCI) leukocytes and leukocyte-platelet aggregates interact with the endothelium at a depth of 100 to 150 μm (lower panel). In contrast, there were few leukocyte-endothelium interactions under normal physiological conditions (that is, 8 h after a sham operation; upper panel). All three axes of the calibration bar are 50 μm.

**Figure 5:**
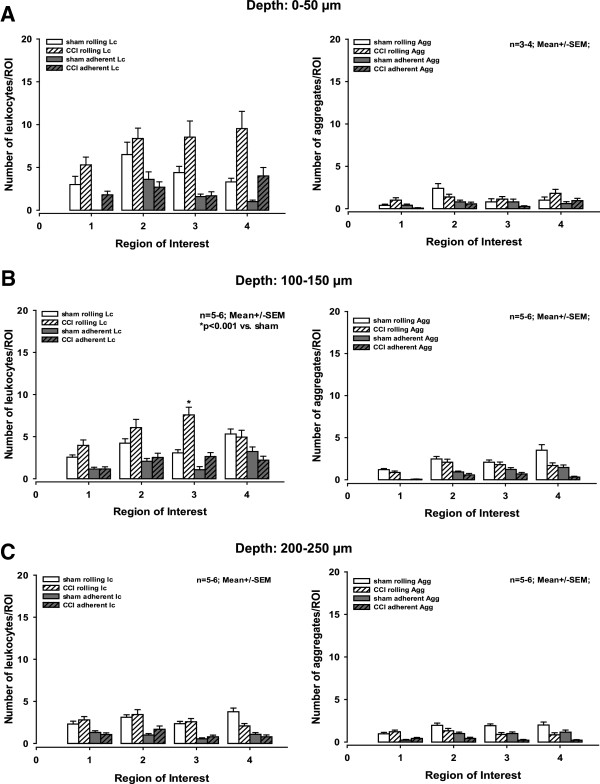
**Quantification of leukocyte-endothelium interaction (LEI) at various depths.** (**A**-**C**) Number of rolling and adherent leukocytes (left) and aggregates (right) at the indicated depths 8 to 10 h after controlled cortical impact (CCI). At each depth, LEI was measured in four standardized regions of interest (ROI). In ROI 4 at 0 to 50 μm and in ROI 3 at 100 to 150 μm, we observed significantly more rolling leukocytes than in sham-operated animals (A and B left, indicated with an asterisk). In all other ROIs at all three depths, we observed no significant differences in LEI between CCI and sham-operated animals.

At depths of 0 to 50 μm and 100 to 150 μm, there was a trend towards an increasing number of rolling leukocytes after trauma relative to sham-operated animals (Figure 4A-D; Figure 5A,B left graphs), which became significant in two ROIs. At a depth of 0 to 50 μm, in ROI 4 we counted 9.5 +/− 2 rolling leukocytes after CCI compared to 4.7 +/− 1.1 in the sham-operated animals (*P < 0.02)*. At a depth of 100 to 150 μm, in ROI 3 we detected 7.7 +/− 0.9 and 4.0 +/− 0.6 rolling leukocytes after CCI and in sham-operated animals, respectively (*P < 0.002*). The average total intravascular volume in an ROI at a depth of 100 to 150 μm was 17,300,000 μm^3^ (Figure 5C left graph).

For all ROI at all depths, the number of adherent leukocytes was less than number of rolling leukocytes, and there were no significant differences between traumatized and sham-operated animals (Figure 5A-C left graphs).

We observed both rolling and adherent aggregates throughout all three levels; however, their numbers were smaller than the number of leukocytes, and there was no significant difference between animals that were subjected to CCI and sham-operated animals (Figure 5A-C right graphs).

### Leukocyte migration into the brain following traumatic brain injury quantified using immunohistochemistry

#### Leukocytes

**Figure 6:**
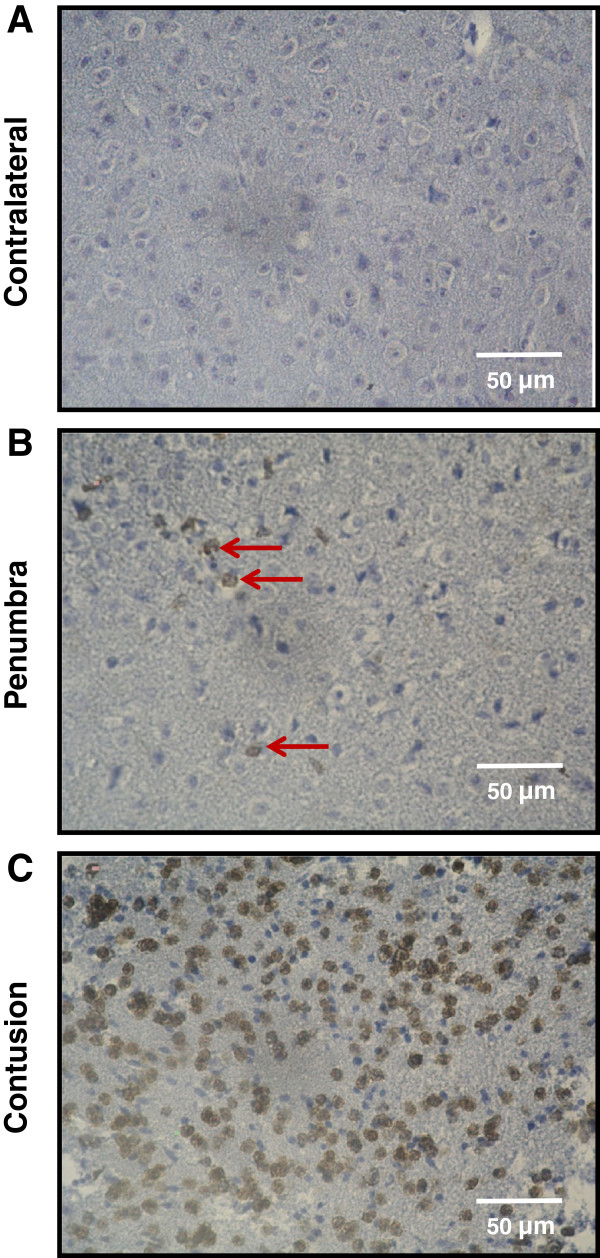
**Representative images of leukocyte migration into the brain 24 h post-controlled cortical impact (CCI). **(**A**) No leukocytes migrated into the contralateral hemisphere. (**B,C**) Following CCI, leukocytes accumulated in the ipsilateral hemisphere. These leukocytes migrated predominantly into the contusion core (**C**), whereas they were nearly absent from the pericontusional penumbra (**B**). The leukocytes in the penumbra are indicated by the red arrows. Leukocytes were labeled using an anti-CD45 antibody.

**Figure 7:**
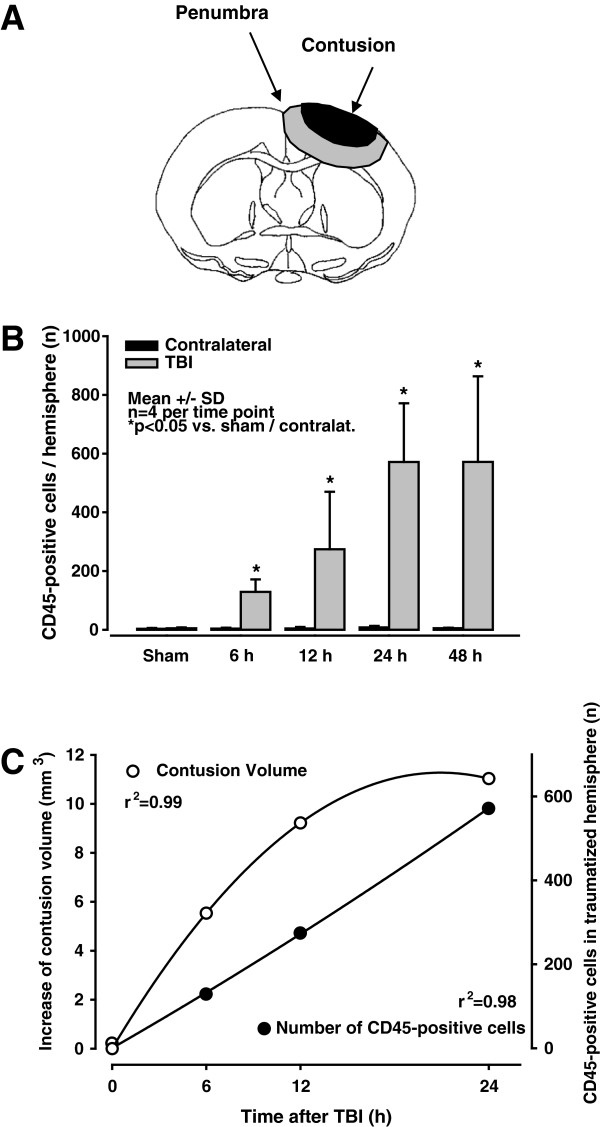
**Leukocyte migration into the brain. **(**A**) Schematic to illustrate the expansion of the secondary lesion. The contusion (black) is necrotic immediately after controlled cortical impact (CCI), and neuronal cell death occurs in the penumbra (gray) throughout the first 24 h. (**B**) Quantification of CD45-positive cells (that is, leukocytes) per hemisphere. The black columns indicate the numbers of leukocytes in the contralateral hemisphere 24 h after CCI. Compared to the contralateral hemisphere, the number of leukocytes in the ipsilateral hemisphere (traumatic brain injury (TBI), gray columns) increased significantly over time, reaching the maximum value at 24 h post-CCI. (**C**) Time course of secondary lesion expansion (measured as necrotic tissue) and leukocyte accumulation in the tissue. The secondary growth of the cortical lesion (open circles) shows a shift to the left as compared to the time course of leukocyte invasion (closed circles). Accordingly, these data suggest that tissue dies first and leukocytes accumulate in the contusion thereafter.

The positive control (see Methods) exhibited staining inside the interfollicular spaces in the spleen. In contrast, we detected no labeled cells in either the negative control or in native brain slices. In the contralateral hemispheres of brains that were removed 24 h post-CCI, which served as controls, we counted 8 +/− 5 leukocytes per hemisphere (Figure 6A, Figure 7B).

At every post-TBI observation time, we measured a significant increase in the number of leukocytes in the injured (ipsilateral) hemisphere relative to the amount in the contralateral side assessed at 24 h post injury (*P < 0.03*). At 24 and 48 h post-injury, a maximum of 571 +/− 200 leukocytes was reached in the ipsilateral hemisphere (Figure 6C, Figure 7B).

#### B lymphocytes

In the positive controls, labeled cells were visible at the edge of the spleen’s follicles, whereas both vaginae periarterialis lymphaticae and the red pulp were unlabeled. Neither the negative control nor the native brain sections contained any stained cells. No B lymphocytes were detected in the brain up to 48 h post-CCI.

#### T lymphocytes

The positive controls exhibited staining of the vaginae periarterialis lymphaticae within the white pulp, whereas the peri-arteriolar spleen noduli and red pulp were unlabeled. Neither the negative control nor native brain sections contained any stained cells. No T lymphocytes were detected in the brain up to 48 h post-CCI.

### Correlation between secondary brain injury and the migration of leukocytes into the brain

**Figure 8:**
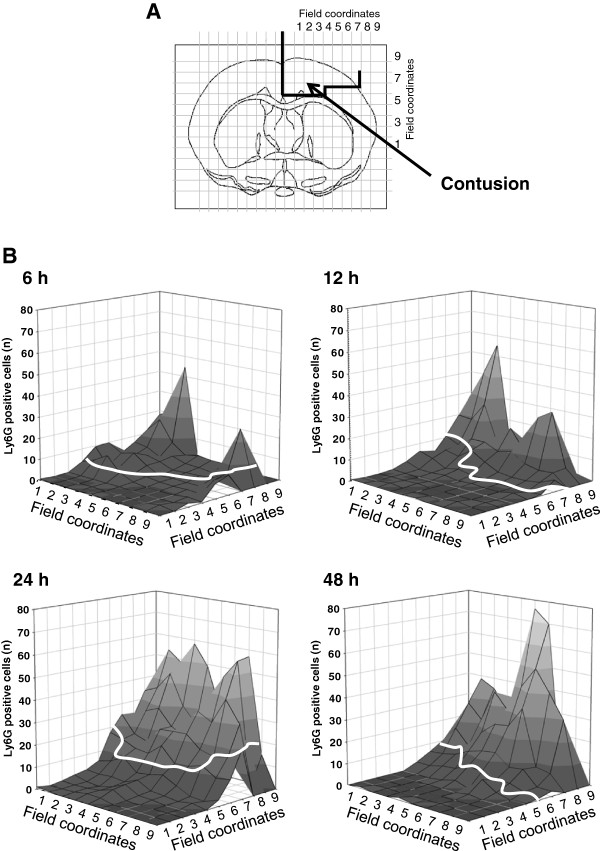
**Post-trauma accumulation of Ly6G-positive granulocytes and monocytes. **(**A**) Histological sections were divided into fields for the precise quantification of leukocytes in relation to the necrotic tissue in the contusion. In the figures in (**B**), the necrotic area is separated from the intact penumbra by the white line. The graphs illustrate the number of Ly6G-positive cells in each field at the indicated times following trauma. Leukocytes appeared in the brain only after neuronal cell death had occurred.

Six hours after CCI, leukocytes were found primarily within the contusion, whereas very few leukocytes were present in the pericontusional penumbra (Figure 6). Both the contusion volume and the number of inflammatory cells in the traumatized hemisphere increased over time, reaching maximum values 24 h after injury (Figure 7B + C). At all of the time points, the inflammatory cells were found predominantly at the center of the contusion (where the tissue was already necrotic), whereas virtually no inflammatory cells were found in the vulnerable penumbra, where the neurons were still alive (Figure 8A-D). This finding indicates that leukocytes accumulated in the tissue only after secondary lesion expansion had occurred. We did not detect any leukocytes in the contralateral hemisphere (Figure 6A; Figure 7B).

### Inhibition of leukocyte adherence using anti-CD18 antibodies

**Figure 9:**
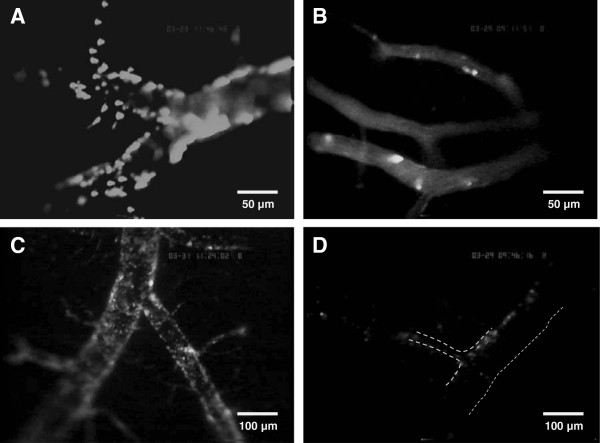
**Representative images of superficial vessels in following administration of anti-CD18 antibodies or control IgG. **(**A**,**B**) Rolling and particularly adhering leukocytes and aggregates in cerebral venules 14 h after controlled cortical impact. The animals received either control IgG (**A**) or anti-CD18 antibodies (**B**). (**C,D**) Cerebral microcirculation at lower magnification (note the difference in scale bases compared with A and B). The animals received either control IgG (**C**) or anti-CD18 antibodies (**D**). After administration of anti-CD18 antibodies the adherence of both leukocytes and aggregates to the venular endothelium was significantly reduced (**B**,**D**).

**Figure 10:**
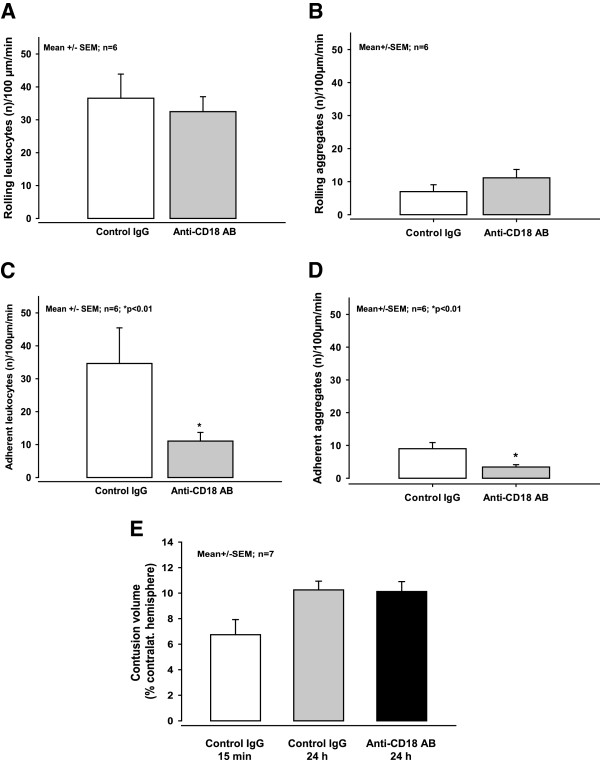
**Effect of anti-CD18 antibodies on leukocyte-endothelium interaction (LEI) and contusion volume. **(**A**) The number of rolling leukocytes in the cerebral venules 14 h after controlled cortical impact (CCI) was not affected by the administration of anti-CD18 antibodies compared to control IgG. (**C**) In contrast, the number of adherent leukocytes was reduced by approximately two-thirds by anti-CD18 antibodies relative to control IgG. (**B,D**) Qualitatively similar results were observed 14 h after CCI with respect to rolling and adhering aggregates in the cerebral venules after the administration of either anti-CD18 antibodies or control IgG. (**E**) Nevertheless, treatment with anti-CD18 antibodies had no effect on the expansion of the secondary lesion, measured 24 h after CCI.

In light of the above results, we investigated the effect of anti-CD18 antibodies on leukocyte adhesion in the venular endothelium at a time in which pronounced LEI occurred, that is, 16 h post-injury. Following the administration of an anti-CD18 antibody (control: IgG), we observed a significant reduction of leukocyte-endothelial interactions in pial microvessels (Figure 9A - D). Quantification of the data revealed no significant difference with respect to rolling leukocytes (Figure 10A). In contrast, the administration of anti-CD18 antibodies reduced the number of adherent leukocytes in the venules by approximately two-thirds the number in animals that received control IgG (11.1 +/− 1.9 versus 33.7 +/− 7.9 adherent leukocytes/100 μm/min, respectively; *P < 0.01*; Figure 2C-F; Figure 10C).

Similar to the results for leukocytes, we observed no significant difference in rolling aggregates following the injection of either anti-CD18 antibody or control IgG (Figure 10B). However, the administration of anti-CD18 antibody reduced the number of adherent aggregates by two-thirds at 16 h post-CCI relative to the control IgG group (3.4 +/− 0.7 versus 9.0 +/− 1.9 adherent aggregates/100 μm/min, respectively; *P < 0.02*; Figure 2C-F; Figure 10D).

### Contusion volume after injection of anti-CD18 antibody or control IgG

Fifteen minutes post-CCI, contusion volume was 6.7 +/− 1.3 mm^3^; this volume is equivalent to 13% of the total volume of the contralateral hemisphere. After 24 h, secondary brain damage increased the contusion volume to 10.3 +/− 0.6 mm^3^ in mice that received control IgG (Figure 10E); a similar increase was observed in mice that received anti-CD18 antibody. 24 h following CCI mice in both groups had contusion volumes that were similar and equivalent to 16% of the volume of the contralateral hemisphere (Figure 10E).

## Discussion

It is well known that TBI can induce an inflammatory reaction [1,27,28]; nevertheless, data supporting a role for blood-borne white blood cells as a causal factor in secondary brain injury following head trauma are highly controversial. There are many possible ways in which leukocytes could contribute to secondary brain damage, including the release of free radicals, the activation of proteases, the production of pro-inflammatory chemokines and cytokines, alterations in cerebral blood flow, and/or increases in vascular permeability [28-31]. These mechanisms could be mediated either via the interaction of leukocytes with the endothelium or via the leukocytes that migrate into the tissue. In the current study, we investigated both possibilities by visualizing leukocytes both in the intravascular space using *in vivo* microscopy and in brain tissue using immunohistochemistry. Of note, all data were obtained from the traumatic penumbra, which is the area of the brain in which secondary brain damage occurs within the first 24 h following brain trauma [18,21,24,32]. Our results demonstrate that both increased LEI and the formation of leukocyte-platelet aggregates are initiated in the microcirculation of the penumbra within the first few hours following TBI. Nevertheless, these effects seem to occur predominantly in superficial vessels, and only to a much lower degree in deeper microvessels. Additionally, leukocytes migrate into the post-TBI brain only after the tissue becomes necrotic. The inhibition of LEI had no effect on secondary lesion expansion following CCI.

### Intravascular leukocyte-endothelium interactions in superficial vessels

Using intravital microscopy, we investigated and quantified LEI up to 13.5 h following CCI. Under normal physiological conditions, LEI was limited to some rolling leukocytes in venules, which is in line with observations published previously by our group [19] and others [33]. Immediately following trauma, however, the number of rolling leukocytes increases significantly and - even more importantly - leukocytes begin to adhere to the venular endothelium. Most interestingly, these events occurred *before* secondary lesion expansion and hence could have potentially mediated secondary brain damage (for example, by disrupting the BBB or by initiating inflammatory cascades).

Elegant studies have suggested a correlation between post-trauma leukocyte accumulation in the brain and secondary brain damage [11,12,29,34,35]. However, in those studies, it was unclear whether the detrimental effect was caused exclusively by leukocyte accumulation or by an associated phenomenon such as leukocyte-endothelium adhesion initiating inflammatory cascades or an up-regulation of the adhesion mediator ICAM-1 and subsequent brain edema formation. Moreover, post-trauma ICAM-1 expression has been correlated with increased permeability of the BBB despite being independent of leukocyte accumulation in the brain [13-16,30,36]. In view of the possibility that ICAM-1 might play a leukocyte-independent role in secondary brain damage [2,31], we used an antibody directed against a structure located on the leukocytes themselves to directly investigate the role of intravascular LEI following TBI. Hence, we used an anti-CD18 antibody that is directed against the beta unit of the lymphocyte function-associated antigen 1 (LFA-1; β-chain CD18 and α-chain CD11a), which binds to ICAM-1 and mediates (among other effects) leukocyte adhesion to the endothelium [2,37,38]. Thus, by blocking the interaction between LFA-1 and ICAM-1, we inhibited leukocyte-endothelium interactions. Using this antibody, we reduced leukocyte adherence by approximately two-thirds compared to an IgG control antibody. However, this did not affect the progression of secondary lesion expansion, indicating that leukocyte adherence to the cerebrovascular endothelium does not play an important role in the pathophysiology of secondary lesion expansion following CCI within the first 24 h. We focused on leukocyte adherence, which has been shown to initiate intracellular signaling and disruption of the BBB [2,39,40]. In contrast, rolling leukocytes interact with the endothelium only very briefly and have not been assigned a role in either initiating inflammatory cascades or opening the BBB.

### Effect of aggregates on secondary brain damage following traumatic brain injury

To date, leukocyte-platelet aggregates have been reported to occur primarily in relation to endothelial stress, for example, due to increased levels of oxidized lipoprotein, inflammation, or diabetes [41-43]. Activated platelets up-regulate their expression of P-selectin, which then binds to its natural ligand, P-selectin-glycoprotein-ligand-1 (PSGL-1), on neutrophils and monocytes [44].

Using intravital microscopy, the formation of leukocyte-platelet aggregates was observed both after subarachnoid hemorrhage (SAH) [45] and after TBI [18]. Following SAH, an antibody directed against P-selectin significantly reduced the formation of leukocyte-platelet aggregates and the adherence of aggregates to the endothelium [45]. Similarly, in our study, inhibiting leukocyte adherence to the endothelium led to a reduction in the adherence of aggregates, which confirms that the aggregates were composed-at least in part-by leukocytes. Nevertheless, unlike the effect of inhibiting P-selectin following SAH, inhibiting LEI did not affect aggregate formation itself. Because aggregates were observed almost exclusively in venules, their effect on the cerebral microcirculation - and in particular, their contribution to vessel occlusion - might not be of primary importance. However, post-TBI microvessel occlusions in tissues outside of the brain have been reported, for example, in the lung [46]. Despite the fact that the reduction of the adherence of aggregates to the venular endothelium did not affect secondary lesion expansion, it remains unclear whether directly inhibiting aggregate formation would have a beneficial systemic/pulmonary effect following TBI. Accordingly, future studies of the role of P-selectin in aggregate formation and the role of post-trauma aggregates both in the brain and in other organs would be needed to clarify these questions.

### Intravascular leukocytes and aggregates in deeper brain levels

Although we also observed rolling and adherent leukocytes and aggregates at a depth of up to 250 μm in the brain using 2-photon microscopy, these events were much less prevalent than in superficial vessels. This effect becomes even more prominent when the average vessel volume is taken into account. The vessel volume investigated in superficial venules (being approximately 85,500 μm^3^) than in deeper regions of the brain (492,100, 173,200, and 125,800 μm^3^ at depths of 0-50, 100-150, and 200-250 μm, respectively).

Several factors may account for this observation. First of all, the diameter of deep vessels (which are primarily capillaries with some arterioles and venules) is much smaller than the diameter of superficial venules. According to the Bernoulli and Venturi Law, a decrease in diameter is accompanied by an increase in blood flow velocity. Therefore, the much faster blood flow velocity in deeper vessels might reduce LEI by increasing shear stress and minimizing cell-cell interactions. Secondly, the post-trauma inflammatory reaction is caused by the contusion itself and is therefore predominantly present in the vessels that drain blood from the site of injury. Hence, leukocyte activation and aggregate formation was scarcely present in arterioles but was present mainly in superficial venules and, to a limited degree, in deeper tissue, most likely in draining capillaries and post-capillary venules.

### Leukocyte migration into vulnerable tissue

Our second aim was to investigate whether leukocytes accumulate in the region of interest (that is, the penumbra) before the tissue becomes necrotic. Because we quantified the expansion pattern of secondary brain damage surrounding the primary contusion at high temporal and spatial resolution [17,21], we could compare the progression of neuronal cell death and leukocyte accumulation within the tissue over time. Following CCI, leukocytes accumulated predominantly in the contusion core. Although significant numbers of granulocytes and monocytes migrated into the tissue during the first 48 h, neither B lymphocytes nor T lymphocytes appeared in the brain. The peak accumulation of leukocytes occurred at 24 to 48 h post-trauma, which is in agreement with previous studies, including a clinical report [47] and several animal experiments using either CCI [15,48] or a weight-drop paradigm [48-50]. Other white blood cells such as monocytes, macrophages, T cells, and B cells appear predominantly 5 to 6 days after trauma [47,50,51], which is in agreement with our results. In contrast, Fee *et al*. reported the presence of activated CD4-positive T cells at the site of traumatic injury within 24 h of aseptic cold injury (ACI) [10]. The authors described a clear correlation between CD4-positive T lymphocytes in the tissue and increased post-traumatic brain damage. This early accumulation of T cells is in contrast to both our results and results from Holmin *et al*. [50] and might be explained - at least in part - by the differences in the trauma models and experimental methods that were used in the respective studies.

In our experiments, leukocytes migrated into the tissue only after neuronal cell death had occurred; thus, their accumulation does not seem to play a role in secondary lesion growth following CCI.

### The role of leukocytes following controlled cortical impact versus other brain injury models

Leukocyte-endothelium adherence and the subsequent migration of leukocytes are known to play a role in secondary brain injury following stroke. This role has been studied extensively, particularly with respect to LFA-1 and Mac-1, which both use the CD18 binding site [6,52-54]. The different result in our study can be most likely attributed to the post-injury differences in the pathophysiology that occur between stroke and TBI and by differences in the processes that are initiated selectively by the mechanical injury that results from CCI [55]. Despite some similarities in the progression of secondary brain damage following both types of injury, the relative importance of individual factors seems to differ. For example, the inflammatory response, including leukocyte-endothelium interactions, clearly plays a more important role in secondary brain injury following stroke than it plays in the wake of TBI [1,55].

Another study reported that leukocyte infiltration into the tissue was correlated to histological outcome after fluid percussion injury (FPI) [11]. The accumulation of monocytes and macrophages within the brain was significantly reduced three days after FPI by the administration of anti-CD11 antibodies, which bind to a fraction of the CD11/CD18 integrin and thereby inhibit leukocyte-endothelium adhesion; most importantly, this reduction in leukocyte accumulation was accompanied by a reduction in lesion volume. However, it remains unclear whether this beneficial effect was due to a reduction in either leukocyte adherence and/or leukocyte migration into the tissue. Again, this discrepancy with our results could be attributed primarily to differences in kinetics initiated by the different experimental models [56]. Firstly, FPI leads to diffuse brain injury that includes hemorrhagic contusions and reaches distant brain regions relative to the primary impact site [56-58]. In contrast, CCI produces a relatively restricted contusion with rapidly developing central necrosis [56,59] in which the diffuse part [32] might be much less important. Hence, in TBI models that produce a much more extensive injury pattern (for example, CCI, in which the maximum lesion volume is reached within 24 h [17]), leukocytes may not contribute significantly to secondary lesion growth compared to models in which the initial injury is less severe and develops over longer periods of time (for example, FPI or ischemic brain injury). This is also supported by the finding that post-CCI leukocyte accumulation in the vulnerable tissue peaks at 24 to 48 h (as shown both in the current study and by others [15,48]), which is 1 to 2 days earlier than the time of peak leukocyte accumulation following FPI [11,51].

### Summary

Following CCI, leukocytes begin to migrate into the injured brain only after neuronal cell death has already occurred. Moreover, leukocytes accumulate predominantly in the contusion core (that is, in tissue that is already necrotic), but barely in the traumatic penumbra, where secondary injury occurs. Inhibiting the adhesion of leukocytes and aggregates to the cerebrovascular endothelium does not reduce the progression of secondary lesion growth. Consequently, our data suggest that blood-borne leukocytes do not mediate secondary lesion expansion following contusional TBI.

## Abbreviations

ACI: aseptic cerebral injury; ACI CBF: cerebral blood flow; BBB: blood–brain barrier; CCI: controlled cortical impact; CD: cluster of differentiation; FITC-dextran: fluorescein isothiocyanate-labeled dextran; FPI: fluid percussion injury; ICAM-1: intercellular adhesion molecule 1; ICP: intracranial pressure; G: immunoglobulin; IVM: intravital fluorescence microscopy; LEI: leukocyte-endothelium interaction; ROI: region of interest; SAH: subarachnoid hemorrhage; TBI: traumatic brain injury.

## Competing interests

The authors declare that they have no competing interests.

## Authors’ contributions

SMS helped designing the project, performed all experiments investigating LEI on the brain surface and the effect of anti-CD18 antibodies on secondary brain damage, analyzed and interpreted the data, provided assistance in designing and performing the experiments investigating deeper regions of the brain, and wrote the manuscript. RZ carried out all experiments determining post-trauma leukocyte accumulation in the tissue and the correlation with secondary brain damage, and analyzed and interpreted the data. NBMG performed all experiments to observe LEI in deeper brain regions and analyzed the data. RT provided assistance in designing and performing the experiments. SWK provided assistance in designing and performing the experiments. NP designed, coordinated and supervised the project, interpreted the data, edited and critically revised the manuscript. All authors read and approved the final manuscript.
